# Perinatal Factors Associated With Mental Health Problems Among Postnatal Women Referred to the University Psychiatry Unit, National Hospital of Sri Lanka

**DOI:** 10.1192/bjo.2025.10158

**Published:** 2025-06-20

**Authors:** Amanda Kasturi Mudali, Chathurie Suraweera, TVM Pieris, MRIF Ariyachandra, AGUS Gunathilaka

**Affiliations:** 1 University Psychiatry Unit, National Hospital of Sri Lanka, Colombo, Sri Lanka; 2 Kent and Medway NHS Social Care and Partnership Trust, Maidstone, United Kingdom; 3 Department of Psychiatry, Faculty of Medicine, University of Colombo, Colombo, Sri Lanka; 4 National Institute of Mental Health, Colombo, Sri Lanka; 5 Lady Ridgeway Hospital for Children, Colombo, Sri Lanka; 6 Colombo North Teaching Hospital, Colombo, Sri Lanka

## Abstract

**Aims:** The postpartum period exerts a profound influence on maternal mental health. This study aimed to describe mental health problems and identify associated perinatal factors in women referred to the University Psychiatry Unit, National Hospital of Sri Lanka (NHSL) during the postnatal period.

**Methods:** A retrospective descriptive cross-sectional study with analytical component was conducted. Data was collected from records of 201 postnatal women randomly selected between 2021–2023 referred from De Soysa Maternity Hospital (DMH) and Castle Street Hospital for Women (CSHW). Women who experienced a perinatal death in their most recent pregnancy were excluded.

**Results:** The mean age of the study population was 30 years. Most were unemployed (73.4%), completed Advanced Level education (39.3%), lived in Colombo district (71.6%), and were married (89.1%) and referred before 5 days postpartum (52%). Among referred patients, 7% had history of depression. Affective symptoms (33.7%) and anxiety (11.2%) were common reasons for referral. The proportion of patients referred for suicidal thoughts was 4.3% and for heroin use was 8%. During assessment 67.2% were diagnosed with a mental illness. Majority had maternal blues (30.4%), depression in 11.8% and postpartum psychosis in 7.4%. Outpatient treatment was offered to 98% of those diagnosed with 81.3% offered pharmacotherapy. It was found that 34.6% defaulted and 21.6% were not sent for review during admission. Postpartum mental illness was significantly associated with gestational age at delivery (χ^2^=8.347, p=0.004), whether baby was admitted to Neonatal Intensive Care Unit or High Dependency Unit (χ^2^=5.424, p=0.02), and spousal support (χ^2^=5.580, p=0.018). No association was found with age, education, past psychiatric history, parity, mode of delivery, or breastfeeding difficulty.

**Conclusion:** The high rate of treatment defaulting indicates a need for improved follow-up and support strategies for women with postpartum mental illness. The identified associated factors of gestational age, neonatal admission status and spousal support can be utilized to design targeted preventative interventions.

